# Transnasal Endoscopic Repair of Unilateral Choanal Atresia in a Young Adult Using a Cross-Over Nasoseptal Flap Technique and a Bioabsorbable Mometasone-Furoate-Eluting Stent: A Case Report

**DOI:** 10.3390/reports9030200

**Published:** 2026-06-25

**Authors:** Athanasios Vlachodimitropoulos, Nicholas S. Mastronikolis, Gerasimos Danielides, Foteini Tsapardoni, Georgios Batsaouras, Spyridon Lygeros

**Affiliations:** 1 Department of Otolaryngology–Head and Neck Surgery, School of Medicine, University of Patras, 26504 Patras, Greece; 2 Department of Ophthalmology, School of Medicine, University of Patras, 26504 Patras, Greece

**Keywords:** choanal atresia, unilateral, adult, transnasal endoscopic surgery, nasoseptal flap, cross-over flap, PROPEL, mometasone furoate, steroid-eluting stent, case report

## Abstract

**Background and Clinical Significance**: Choanal atresia is a rare congenital obstruction of the posterior nasal aperture, with an estimated incidence of one in 5000 to one in 8000 live births. Bilateral disease typically presents as a neonatal emergency, whereas unilateral disease is more frequent and may remain undiagnosed for years or decades, presenting in adolescence or adulthood with chronic unilateral nasal obstruction and ipsilateral mucopurulent rhinorrhoea. Optimal surgical management remains debated, particularly with regard to mucosal-flap reconstruction and the choice of postoperative stent. **Case Presentation:** A 22-year-old male was referred for chronic left-sided nasal obstruction, persistent ipsilateral mucopurulent rhinorrhoea and reduced ipsilateral olfaction. Nasal endoscopy and high-resolution computed tomography demonstrated an isolated, non-syndromic, mixed bony–membranous left choanal atresia. The patient underwent transnasal endoscopic choanoplasty with posterior septectomy and removal of the atretic plate and posterior vomer. An ipsilateral superiorly based septal mucoperichondrial flap was raised first and later transposed over the sphenoid rostrum; following drilling, the contralateral septal mucosa was approached and incised horizontally to generate a superior and an inferior leaflet, which were rotated to cover the corresponding portions of the residual posterior septal ridge. A bioabsorbable mometasone-furoate-eluting sinus implant (PROPEL^®^, Medtronic) was deployed across the neo-choana. The follow-up endoscopy at two months demonstrated a widely patent, well-mucosalized neo-choana with complete resolution of symptoms. **Conclusions:** Transnasal endoscopic posterior septectomy combined with mucosal-flap reconstruction and a bioabsorbable steroid-eluting stent is a technically feasible and biologically rational approach to adult unilateral CA. To our knowledge, this is among the first reports describing the off-label intraoperative use of a PROPEL^®^ stent in a young adult with isolated unilateral choanal atresia.

## 1. Introduction and Clinical Significance

Choanal atresia (CA) is a rare congenital malformation of the posterior nasal aperture, defined as the developmental absence of communication between the nasal cavity and the nasopharynx. It was first described by Roederer in 1755 [1]. The reported incidence is approximately one in 5000 to one in 8000 live births, with a slight female predominance and an approximate 2:1 ratio of unilateral to bilateral disease; right-sided unilateral disease is more common than left-sided [2,3]. Although several embryological hypotheses have been proposed—including persistence of the buccopharyngeal membrane, persistence of the nasobuccal membrane of Hochstetter, abnormal mesodermal adhesions, and misdirection of neural-crest-cell migration as proposed by Hengerer and Strome—the precise mechanism remains incompletely understood [4,5,6]. *CHD7* mutations are the genetic basis of the strong association with CHARGE syndrome, which is much more frequently encountered in bilateral CA [7,8].

Contemporary high-resolution computed tomography (CT) has substantially refined the anatomical understanding of CA. In their seminal series, Brown and colleagues reported that 71% of cases are mixed bony–membranous and 29% are pure bony, with no true purely membranous case observed; thickening of the posterior vomer and medial bowing of the medial pterygoid plate are common features that have direct surgical implications [9]. Adequate widening of the neo-choana therefore typically requires drilling of both the vomer and the lateral pterygoid component rather than simple membrane perforation [10].

Clinical presentation differs sharply by laterality. Bilateral CA manifests at birth as cyclical respiratory distress and cyanosis paradoxically relieved by crying—a neonatal emergency, since neonates are obligate nasal breathers [3]. Unilateral CA, in contrast, is often paucisymptomatic and may evade diagnosis for years or decades, presenting with chronic unilateral nasal obstruction, persistent ipsilateral mucoid or mucopurulent rhinorrhoea, recurrent rhinosinusitis and ipsilateral hyposmia [11,12]. Diagnosis rests on (i) failure to pass a 5–6-French catheter beyond the posterior choana, (ii) nasal endoscopy and (iii) high-resolution CT with axial and coronal reconstructions, which is the gold standard for surgical planning [9,11].

Surgical management has evolved markedly. The transpalatal approach offers excellent exposure but is associated with significant morbidity, including palatal fistula and disturbed mid-facial growth in children, and has been progressively abandoned [13]. The transseptal approach was proposed as an intermediate alternative [14]. Since Stankiewicz first described the technique in 1990, the transnasal endoscopic approach has become the gold standard, supported by powered instrumentation and refined mucosal-flap techniques, with primary success rates of 67–88% in pooled series [15,16]. Several mucosal-flap variants have been described—including the nasal septal cross-over flap of Stamm and Pignatari, the single side-hinged flap, and the recent two-center cross-over septal series of Adegboyega and colleagues—all sharing the principle of resurfacing exposed bone with viable, vascularized mucosa to prevent circumferential scarring and restenosis [17,18,19]. Adjuncts to reduce restenosis have included topical mitomycin-C and stenting; recent meta-analyses have suggested that conventional silastic or endotracheal-tube stenting may not improve patency and may increase complication rates [20,21,22].

In recent years, off-label intraoperative use of bioabsorbable, mometasone-furoate-eluting sinus implants (PROPEL^®^, Medtronic, Dublin, Ireland; originally Intersect ENT, approved for the ethmoid and frontal sinus ostia following functional endoscopic sinus surgery) has been proposed as a way to combine mechanical patency with sustained, localized corticosteroid release, while obviating the need for stent-removal procedures [23,24]. The published experience in CA, although limited, is uniformly favorable [25,26,27,28,29]. Almost all reported cases have been pediatric; the use of this technology in the adult population is exceedingly rare [25,28,30].

**Clinical significance:** Adult presentation of unilateral CA is uncommon and may initially be misdiagnosed as chronic rhinosinusitis or septal pathology. We present a 22-year-old male managed with transnasal endoscopic posterior septectomy, cross-over-type nasoseptal flap reconstruction and intraoperative deployment of a bioabsorbable mometasone-furoate-eluting stent, with the aim of illustrating both the diagnostic pathway and a contemporary, mucosa-sparing reconstructive strategy in the adult population, in whom this combination of techniques has been very rarely reported.

## 2. Case Presentation

### 2.1. Patient Information and Clinical Findings

A 22-year-old male of Caucasian (Greek) ethnicity, with no medical or surgical history of note, no regular medication and no known drug allergies, was referred to our Department of Otolaryngology—Head and Neck Surgery. He reported lifelong left-sided nasal obstruction that had become increasingly bothersome during adolescence, accompanied by persistent left-sided thick mucoid to mucopurulent rhinorrhoea, intermittent left-sided facial pressure with episodes consistent with recurrent acute rhinosinusitis, mild snoring, and a subjective decrease in olfaction predominantly on the left side. He denied epistaxis, dysphagia or any neurological symptom. There was no relevant family history, no maternal exposure to antithyroid drugs or other known teratogens, and no features of CHARGE syndrome (no coloboma, no congenital heart disease, no ear anomalies, no genital hypoplasia, normal growth and development).

Anterior rhinoscopy revealed thick mucoid secretions in the left nasal cavity. Cold-spatula misting was reduced on the left. Rigid nasal endoscopy with 4 mm 0° and 30° Hopkins-rod telescopes (Tuttlingen, Germany) demonstrated patent right-sided choana with normal nasopharyngeal anatomy, while on the left a complete obliteration of the posterior choana was observed at the level of the posterior end of the inferior turbinate, with a thickened, medially bowed lateral wall and a thickened posterior septum. Failure to pass a 6-French suction catheter through the left nasal cavity into the nasopharynx confirmed mechanical obstruction. Otoscopy and oropharyngeal examination were normal. Ophthalmological and cardiological screening were unremarkable.

### 2.2. Diagnostic Assessment

High-resolution CT of the paranasal sinuses with axial and coronal reformations was performed. Imaging confirmed a left-sided choanal atresia with a mixed bony–membranous atretic plate, thickening of the posterior vomer and medial bowing of the left medial pterygoid plate, with consequent narrowing of the posterior left nasal cavity, in keeping with the “mixed” pattern described by Brown and colleagues [9]. The right choana, paranasal sinuses, skull base, sphenoid sinus and orbits were unremarkable. No additional craniofacial anomaly was identified (Figure 1).

The differential diagnosis of unilateral nasal obstruction with persistent mucopurulent discharge in a young adult included antrochoanal polyp, sinonasal inverted papilloma, post-traumatic posterior synechiae, nasopharyngeal mass and acquired choanal stenosis (post-inflammatory, post-surgical or post-radiation). Endoscopy and CT excluded these alternatives and confirmed isolated, non-syndromic congenital unilateral CA.

### 2.3. Therapeutic Intervention

After multidisciplinary discussion and informed consent, the patient was scheduled for elective transnasal endoscopic choanoplasty.

**Anesthesia and preparation.** The procedure was performed under general anesthesia with orotracheal intubation. After topical decongestion of both nasal cavities with cottonoid pledgets soaked in 1:1000 adrenaline, the operative field was infiltrated with 1:100,000 adrenaline along both sides of the nasal septum.

**Endoscopic exposure and ipsilateral flap elevation.** Using a 4 mm, 0° rigid endoscope, the left atretic plate was visualized. As a first step, on the ipsilateral (left) side, a superiorly based septal mucoperichondrial flap was outlined and elevated over the posterior septum. The flap was raised in a subperichondrial/subperiosteal plane with a Cottle elevator and reflected superiorly, exposing the underlying bony posterior septum and atretic plate while preserving its superior pedicle. This flap was reserved for later coverage of the sphenoid rostrum.

**Posterior septectomy and removal of the atretic plate.** With the ipsilateral mucosa atraumatically retracted, the posterior bony septum (vomer) was initially thinned using a powered microdrill fitted with a 3 mm diamond burr under continuous saline irrigation. Once adequately thinned, the remaining bony posterior septum was removed piecemeal using cutting forceps, allowing controlled, precise bone removal. The membranous and mucosal components of the atretic plate were subsequently resected using a 4 mm straight microdebrider blade, which permitted clean debulking of the soft-tissue element while minimizing mucosal trauma to the surrounding preserved flaps. The medial aspect of the medial pterygoid plate was then conservatively thinned with the diamond burr to widen the lateral component of the neo-choana, with care taken to preserve the integrity of the skull base superiorly and the hard palate inferiorly. A wide, common posterior cavity continuous with the nasopharynx was thereby created. A residual mucosal defect remained on the lateral nasal wall, where the atretic plate had been drilled and where lateral wall mucosa could not be elevated as a flap; this raw bony surface was left to re-epithelialize by secondary intention, in line with established practice when the alternative would entail flap inset under tension [17,19].

**Contralateral mucosal-flap design and inset.** Once the posterior septectomy had been completed and the contralateral (right-sided) septal mucosa was directly accessible, a horizontal incision was made along its midportion—thereby generating a posteriorly pedicled superior leaflet and a posteriorly pedicled inferior leaflet. The previously raised ipsilateral superior septal flap was then advanced posteriorly to drape over the sphenoid rostrum, eliminating exposed bone in this area. The superior leaflet was rotated medially and superiorly to cover the superior aspect of the residual bony septal ridge; the inferior leaflet was rotated medially and inferiorly to cover the inferior aspect of the ridge and the floor of the neo-choana (Figure 2).

**Stenting.** A standard Medtronic PROPEL^®^ bioabsorbable, self-expanding, mometasone-furoate-eluting sinus implant (poly-L-lactide-co-glycolide [PLG] copolymer scaffold containing 370 µg of mometasone furoate, designed for sustained release over approximately 30 days) was deployed across the newly fashioned left neo-choana under endoscopic guidance, providing both mechanical patency and a localized reservoir of corticosteroid [23,24]. The implant was positioned with its longitudinal axis perpendicular to the anteroposterior (longitudinal) axis of the nasal cavity—that is, lying transversely across the neo-choana—so that the self-expanding scaffold splinted the aperture in the coronal plane and apposed the rotated mucosal flaps against the posterior septal ridges and neo-choanal margins. No trimming or customization of the device was performed, and no additional fixation or suturing was required; radial self-expansion alone retained the implant in position. The stent was used off-label in this anatomical site, in keeping with previously reported applications in CA [25,26,27,28,29].

**Closure and immediate postoperative care.** No nasal packing was used. The total operative time was approximately 90 min, with minimal blood loss. The patient was extubated uneventfully and discharged on postoperative day one.

### 2.4. Postoperative Care and Follow-Up

Postoperative management included high-volume isotonic saline nasal irrigations 3–4 times daily, started 24 h after surgery, a short course of oral broad-spectrum antibiotic (amoxicillin–clavulanic acid for 7 days), avoidance of forceful nose-blowing for 4 weeks and scheduled outpatient nasal endoscopies at 1 week, 1 month, 2 months and 6 months. The 7-day course of oral amoxicillin–clavulanic acid was prescribed on a targeted rather than routine basis, given the patient’s long-standing ipsilateral mucopurulent rhinosinusitis together with the extensive intranasal bone work and mucosal-flap reconstruction performed in a chronically colonized field.

At the one-month follow-up, rigid nasal endoscopy demonstrated a patent neo-choana with early but satisfactory mucosal healing. The PROPEL^®^ implant had fully bioabsorbed and was no longer visible. No device-related complications were observed: there was no implant migration into the nasopharynx or airway, no foreign-body or hypersensitivity reaction and no excessive granulation attributable to the implant. The septal flap leaflets were beginning to establish mucosal coverage of the posterior septal ridge both superiorly and inferiorly (Figure 3A).

At the two-month follow-up, rigid nasal endoscopy demonstrated a wide, well-mucosalized left neo-choana with smooth re-epithelialization of the lateral nasal wall, no synechiae, no significant granulation tissue and only minimal mucoid secretion (Figure 3B). The patient reported complete resolution of left-sided nasal obstruction and rhinorrhoea, normalization of subjective olfaction and absence of further sinusitis episodes.

### 2.5. Patient Perspective and Informed Consent

The patient reported a marked improvement in his quality of life, with the disappearance of chronic left-sided nasal symptoms and a return to unobstructed nasal breathing. He expressed satisfaction with the minimally invasive nature of the procedure and the absence of pain related to traditional stents. Written informed consent for the procedure and for the publication of de-identified clinical images and details was obtained from the patient.

## 3. Discussion

Adult presentation of unilateral CA, although uncommon, is a recognized clinical entity [11,12,31]. In a multi-institutional Canadian study of 215 patients, the mean age at initial repair was 0.8 months for bilateral CA and 48.6 months for unilateral CA, but isolated case reports document first presentation in the third, fourth, sixth and even seventh decades of life [11,31,32,33]. Our 22-year-old patient falls within this clinical spectrum. Importantly, isolated unilateral CA has a much weaker association with syndromic disease than bilateral CA, in which up to 50% of cases harbour an additional anomaly, most frequently CHARGE syndrome [7,8]. A formal multisystem evaluation excluding coloboma, congenital heart disease, ear anomalies and genital hypoplasia therefore remains good practice in any newly diagnosed case and was performed in our patient with negative results.

High-resolution CT of the paranasal sinuses is the gold-standard imaging modality and provides the three pieces of information that determine the surgical strategy: (i) the nature of the atretic plate, (ii) the thickness of the posterior vomer, and (iii) the degree of medial bowing of the lateral nasal wall [9,11]. Our patient demonstrated all three of the typical features of mixed CA, requiring not only perforation of the obstructing plate but also drilling of the vomer and conservative thinning of the lateral pterygoid component to create a stable neo-choana.

The transnasal endoscopic approach has progressively become the standard of care because it offers direct visualization, allows the use of powered instrumentation, preserves palatal anatomy and is associated with primary success rates of 67–88% in pooled series [15,16]. In adult unilateral CA, several recent series confirm excellent and durable outcomes after endoscopic posterior septectomy [12,31].

Restenosis is the principal long-term complication of any choanoplasty. Historical restenosis rates of 9–36% have been reported for non-flap or flapless techniques [3,16]. The pathophysiology involves circumferential exposure of denuded bone at the neo-choana, which heals by secondary intention with granulation and contractile fibrosis, resulting in concentric scar formation. Mucosal preservation and rotation/cross-over flaps act as a definitive solution to this problem because they cover the raw bony rim with viable, vascularized mucosa, interrupt the circumferential ring of granulation tissue and accelerate physiological re-epithelialization [17,18,19,29]. Multiple mucosal-flap variants have been described—including the original nasal septal cross-over flap of Stamm and Pignatari, the single side-hinged flap of Saraniti and colleagues, the mirrored L-shaped septonasal flap of Wang and colleagues, and the recent multicenter cross-over septal flap series of Adegboyega and colleagues [17,18,19,29].

The strategy adopted in our patient is conceptually closest to the cross-over septal flap, modified for unilateral disease. The ipsilateral superior septal flap was raised first, in order to expose the underlying bone and allow safe drilling without compromising contralateral mucosa; only after the bony work had been completed and the contralateral septal mucosa was directly accessible from the neo-choana was the contralateral mucosa incised. A single horizontal incision along the midportion of the contralateral mucosa generated a posteriorly pedicled superior leaflet and a posteriorly pedicled inferior leaflet, both of which were rotated medially to cover the corresponding portions of the residual ridge. The previously raised ipsilateral superior flap was then transposed over the sphenoid rostrum, where granulation is otherwise particularly common. This sequence ensures that the most critical surfaces—the sphenoid rostrum, the resected posterior septum and the floor of the neo-choana—are covered by viable mucosa at the conclusion of surgery, while the lateral nasal wall is purposely spared from flap manipulation. The lateral nasal wall mucosal defect, where the atretic plate had been drilled, was left to re-epithelialize by secondary intention; in our patient, healing in this region was uneventful, as documented by the two-month endoscopic appearance (Figure 3). This approach avoids the risk of poorly vascularized flap inset under tension that would result from forced lateral wall coverage [17,19].

Postoperative stenting has historically aimed to maintain mechanical patency of the neo-choana during early healing, but is associated with well-documented complications including alar and columellar pressure necrosis, infection, granulation tissue formation, foreign-body reaction and patient discomfort, as well as the need for a second anesthetic for stent removal [20,21]. Two systematic reviews and meta-analyses have shown that conventional stenting does not improve patency and may increase complication rates compared with stentless flap techniques [20,21,22]. Topical mitomycin-C, originally introduced as an anti-fibrotic adjunct, has yielded conflicting results [34,35]. Within this context, the off-label use of bioabsorbable, mometasone-furoate-eluting sinus implants (PROPEL^®^, Medtronic, originally Intersect ENT) is mechanistically attractive: the device is a self-expanding PLG scaffold that releases 370 µg of mometasone furoate locally over approximately 30 days and is fully bioabsorbed without the need for surgical removal [23,24,36]. PROPEL^®^ received U.S. Food and Drug Administration approval in 2011 for use in the ethmoid sinus following functional endoscopic sinus surgery, with subsequent indications for the frontal sinus [36,37]. The combination of mechanical splinting and sustained, localized corticosteroid release addresses the two principal drivers of restenosis (mucosal collapse and inflammatory granulation) in a single device.

The published experience of PROPEL^®^ in CA is small but consistently positive. Bangiyev and colleagues first described the use of mometasone-furoate-eluting stents in three pediatric patients (aged 4 days to 16 years; bilateral and unilateral; bony, membranous and mixed types), with no restenosis at 12 months [25]. Meleca and colleagues subsequently reported a case series in which steroid-eluting stents reduced the need for repeat general anesthesia [26]. Wilcox and colleagues described a single-institution case series confirming favorable outcomes both in primary repair and in restenosis [27]. Wang and colleagues compared the septonasal flap technique with bioabsorbable steroid-eluting stents versus silicone stents and demonstrated reduced operative time, hospital stay, number of procedures and restenosis with the bioabsorbable device [29]. Skaribas and colleagues reported their use specifically in pediatric CA restenosis [28]. Galletti and colleagues most recently described a customized PROPEL^®^ insertion technique in a seven-day-old neonate with bilateral CA [30]. Nearly all published cases have been pediatric: Bangiyev’s 16-year-old (unilateral mixed type) is the oldest patient previously reported in detail [25]. Our 22-year-old patient therefore extends the experience with PROPEL^®^ into the young adult population and, to our knowledge, represents the first description of a cross-over-type nasoseptal flap reconstruction combined with a PROPEL^®^ bioabsorbable stent in an adult with isolated unilateral CA.

Several limitations are inherent to a single case report. First, follow-up is short (two months); durable patency without symptomatic restenosis requires 12–24 months of endoscopic surveillance, particularly given that most restenoses occur within the first six months. Second, the contribution of each individual technical element (posterior septectomy, sequenced ipsilateral and contralateral flap inset, steroid-eluting stent) cannot be disaggregated from a single observation. Third, off-label use of the PROPEL^®^ device has cost implications and is not yet supported by randomized data in CA; an ongoing single-blind randomized controlled trial is expected to clarify this question [38]. Finally, the residual lateral wall mucosal defect is a known weakness of cross-over-type techniques; alternative reconstructions might be considered in selected cases.

## 4. Conclusions

Adult-onset unilateral CA, while uncommon, should be actively considered in young adults with chronic unilateral nasal obstruction and ipsilateral mucopurulent rhinorrhoea. CT-confirmed diagnosis allows mucosa-sparing transnasal endoscopic posterior septectomy, which is now the gold-standard surgical approach. The combination of a sequenced flap reconstruction with a bioabsorbable mometasone-furoate-eluting sinus implant (PROPEL^®^) is a logical, biologically rational adjunct: it preserves vascularized mucosa where it most matters, eliminates the morbidity of traditional stent removal and delivers sustained localized corticosteroid to the healing field. In our 22-year-old patient, this strategy yielded a widely patent, well-mucosalized neo-choana at two months with complete symptom resolution. Larger prospective studies, ideally randomized, are needed to confirm whether bioabsorbable steroid-eluting stents should become a routine adjunct to mucosal-flap-based endoscopic choanoplasty, particularly in adults with unilateral disease.

## Figures and Tables

**Figure 1:**
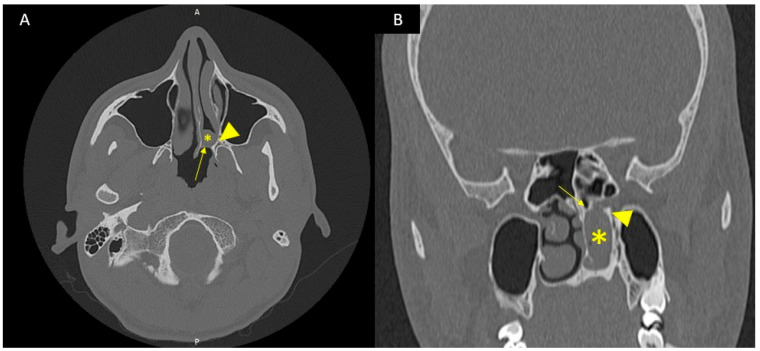
Preoperative high-resolution CT of the paranasal sinuses (bone window). (**A**) Axial view at the level of the posterior choanae demonstrating complete obliteration of the left posterior choana by a mixed bony–membranous atretic plate (arrow), with thickening and medial bowing of the left medial pterygoid plate (arrowhead); retained secretions in the obstructed left nasal cavity are noted (asterisk); the right choana is patent. (**B**) Coronal reformation demonstrating the superior-to-inferior extent of the left choanal atresia (asterisk) with a markedly thickened posterior vomer (arrow), medial bowing of the left medial pterygoid plate (arrowhead) and asymmetric narrowing of the left posterior nasal cavity.

**Figure 2:**
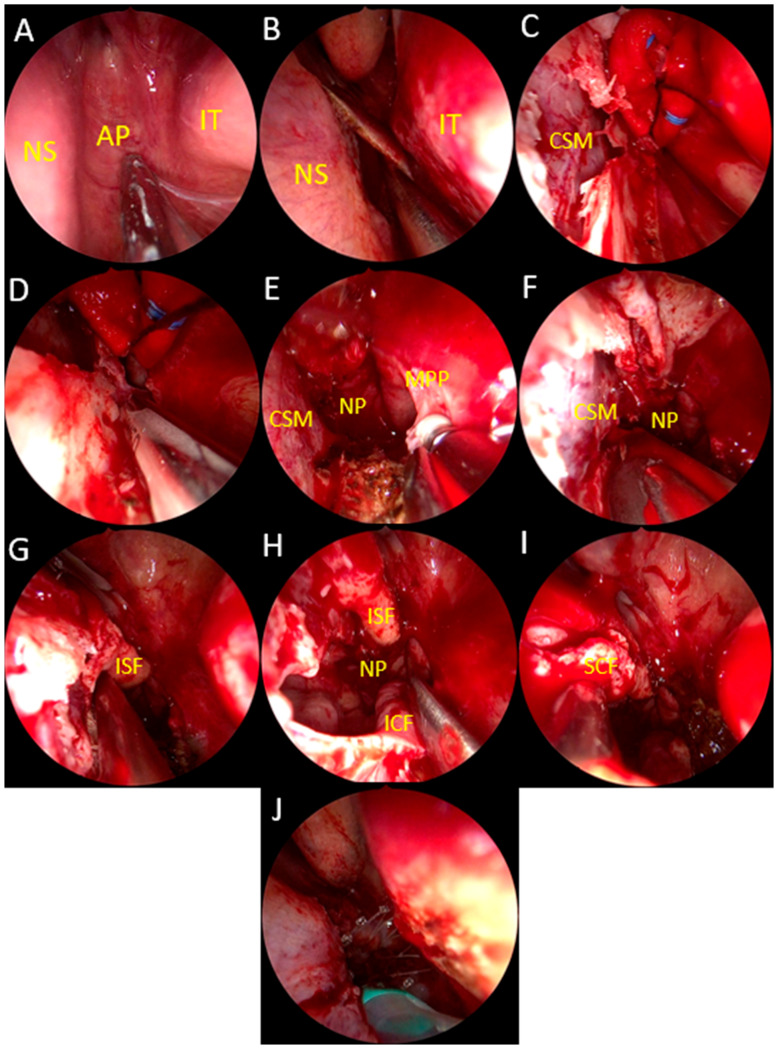
Intraoperative endoscopic views of the transnasal endoscopic choanoplasty (0° rigid endoscope). (**A**) Preoperative endoscopic view of the left nasal cavity demonstrating the atretic plate (AP) medial to the inferior turbinate (IT), with the nasal septum (NS) visible medially. (**B**) Elevation of the ipsilateral superiorly based septal mucoperichondrial flap (ISF) as the first surgical step, raised in a subperichondrial plane and reflected superiorly to expose the underlying bony posterior septum and AP. (**C**) Following partial posterior septectomy, the contralateral septal mucosa (CSM) becomes visible through the newly created opening. (**D**) Resection of the membranous component of the AP using a microdebrider, achieving clean debulking of the soft-tissue element while preserving the surrounding mucosal flaps. (**E**) Diamond burr drilling of the medial pterygoid plate (MPP), widening the lateral dimension of the neo-choana; the nasopharynx (NP) and CSM are visible through the enlarged aperture. (**F**) A single horizontal incision is made to the CSM, generating a superior and an inferior leaflet without any vertical incision. (**G**) The ISF is transposed posteriorly and superiorly and draped over the sphenoid rostrum. (**H**) The inferior contralateral flap (ICF) is rotated medially and inferiorly, covering the inferior aspect of the residual posterior septal ridge. (**I**) The superior contralateral flap (SCF) is rotated medially to cover the superior aspect of the residual posterior septal ridge, completing the mucosal reconstruction of the neo-choana. (**J**) Final placement of the PROPEL^®^ bioabsorbable mometasone-furoate-eluting stent across the neo-choana. **Abbreviations:** AP, atretic plate; IT, inferior turbinate; NS, nasal septum; CSM, contralateral septal mucosa; MPP, medial pterygoid plate; NP, nasopharynx; ISF, ipsilateral septal flap; ICF, inferior contralateral flap; SCF, superior contralateral flap.

**Figure 3:**
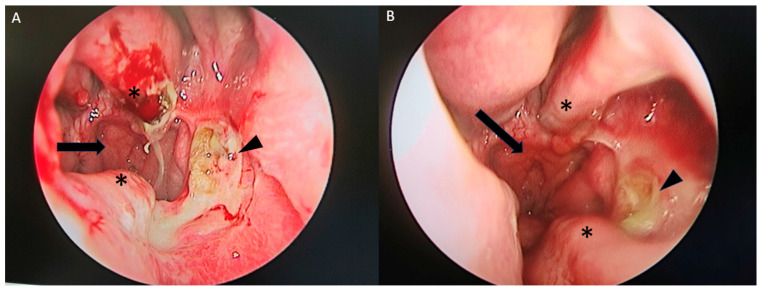
Postoperative rigid nasal endoscopy of the neo-choana (0° rod telescope). (**A**) One-month postoperative view demonstrating a patent neo-choana (arrow) with early mucosal healing. The septal flaps show initial coverage of the posterior septal ridge remnants superiorly and inferiorly (asterisks). The mucosal defect on the posterior lateral nasal wall can be seen (arrowhead). (**B**) Two-month postoperative view demonstrating a widely patent neo-choana (arrow) with no evidence of restenosis or synechiae. The septal flaps demonstrate smooth, well-vascularized mucosal coverage of the posterior septal ridge remnants both superiorly and inferiorly (asterisks). The lateral nasal wall shows progressive re-epithelialization of the area where the atretic plate was drilled, with no significant granulation tissue (arrowhead).

## Data Availability

The data that support the findings of this study are available upon request from the corresponding author. The data are not publicly available due to privacy or ethical restrictions.

## Abbreviations

The following abbreviations are used in this manuscript:

CA	Choanal Atresia
CHARGE	Coloboma, Heart defects, Atresia Choanae, Retardation, Genital abnormalities, Ear abnormalities
CT	Computed Tomography
PLG	Poly-L-lactide-co-glycolide
